# Weinsäure – Bestimmung von L-(+)- und D-(–)-Weinsäure in der Luft am Arbeitsplatz mittels Ionenchromatographie (IC): Luftanalysen-Methode

**DOI:** 10.34865/am52683d10_2or

**Published:** 2025-06-30

**Authors:** Ulrich Prott, Claus-Peter Maschmeier, Ralph Hebisch, Andrea Hartwig, MAK Commission

**Affiliations:** 1 Bundesanstalt für Arbeitsschutz und Arbeitsmedizin (BAuA) Friedrich-Henkel-Weg 1–25 44139 Dortmund Deutschland; 2 Land Sachsen-Anhalt Gebrüder-Bethmann-Str. 18 06862 Dessau-Roßlau Deutschland; 3 Institut für Angewandte Biowissenschaften. Abteilung Lebensmittelchemie und Toxikologie. Karlsruher Institut für Technologie (KIT) Adenauerring 20a, Geb. 50.41 76131 Karlsruhe Deutschland; 4 Ständige Senatskommission zur Prüfung gesundheitsschädlicher Arbeitsstoffe. Deutsche Forschungsgemeinschaft, Kennedyallee 40, 53175 Bonn, Deutschland. Weitere Informationen: Ständige Senatskommission zur Prüfung gesundheitsschädlicher Arbeitsstoffe | DFG

**Keywords:** Weinsäure, Luftanalysen, Analysenmethode, Arbeitsplatzmessung, Gefahrstoff, Ionenchromatographie, Leitfähigkeitsdetektion, IC, Glasfaserfilter, Flüssigdesorption, tartaric acid, air analyses, analytical method, workplace measurement, hazardous substance, ion chromatography, conductivity detector, IC, glass fibre filter, liquid desorption

## Abstract

The working group “Air Analyses” of the German Senate Commission for the Investigation of Health Hazards of Chemical Compounds in the Work Area (MAK Commission) developed and verified the presented analytical method. It is used to determine the levels of L-(+)-tartaric acid [87-69-4] and D-(–)-tartaric acid [147-71-7] (occurring as inhalable particles) individually or as a racemic mixture [133-37-9] that occur in the workplace air. The method covers concentrations in the range from one tenth up to twice the current Occupational Exposure Limit Value (OELV) of 2 mg/m^3^ (inhalable fraction). The method is also suitable for measuring the short-term exposure limit (STEL; excursion factor 2) for the inhalable fraction. Samples are collected by drawing a defined volume of air through a glass fibre filter, which is inserted in a GSP sampling system, using a flow regulated pump at a volumetric flow rate of 3.5 l/min. Exposure during the shift is measured with a sampling period of 2 hours and the short-term exposure with a period of 15 minutes. Tartaric acid deposited on the glass fibre filter is extracted with the IC eluent and analysed by ion chromatography using conductivity detection. The quantitative determination is based on multiple-point calibrations with external standards. A relative limit of quantification (LOQ) of 0.00043 mg/m^3^ is obtained for an air sample volume of 420 litres. As the LOQ for a sample volume of 52,5 litres is 0.0034 mg/m^3^, the STEL can also be measured. The recovery is 100–104% and the expanded uncertainty is 19–21% for a 2-hour sampling and 20–21% for a 15-minute sampling.

**Table:** 

**Methodennummer**	1
**Anwendbarkeit**	Luftanalyse
**Analyt. Messprinzip**	Ionenchromatographie mit Leitfähigkeitsdetektion (IC)

## 1 Kenndaten des Verfahrens

**Table:** 

**Präzision:**	Standardabweichung (rel.):	*s* = 0,67–0,94 %
	Erweiterte Messunsicherheit:	*U* = 19–21 %
	in einem Bereich von 0,1–2 mg/m^3^ und n = 6	
**Bestimmungsgrenze:**	0,018 mg/l in der Messlösung	
	0,00043 mg/m^3^ bei einem Probeluftvolumen von 0,42 m^3^ und einer Probenahmedauer von 2 Stunden 0,0034 mg/m^3^ bei einem Probeluftvolumen von 0,0525 m^3^ und einer Probenahmedauer von 15 Minuten	
**Wiederfindung:**	*η* = 100–104 %	
**Probenahmeempfehlung:**	Probenahmedauer:	2 h
	Probeluftvolumen:	420 l
	Volumenstrom:	3,5 l/min
	Für Kurzzeitmessungen:	15 min; 52,5 l

## 2 Stoffbeschreibung

### Weinsäure

**Abb.1:**
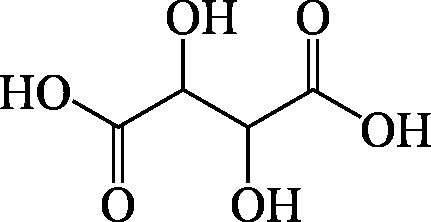
Strukturformel von Weinsäure (ohne Stereoisomerie)

Von den drei Stereoisomeren der Weinsäure [526-83-0] (siehe Abbildung 1, auch 2,3-Dihydroxybutandisäure, 2,3-Di­hydroxybernsteinsäure, Traubensäure (Racemat), Threarsäure, Weinsteinsäure, E334 genannt) kommen die Isomere D-(–)-Weinsäure [147-71-7] und *meso*-Weinsäure [147-73-9] sowie das racemische Gemisch aus L-(+)- und D-(–)-Weinsäure [133-37-9], das Traubensäure genannt wird, in der Natur kaum vor. Natürlich wird fast ausschließlich L-(+)-Weinsäure [87-69-4] gebildet. Die Salze der Weinsäure heißen Tartrate.

Weinsäure ist ein weißer, geruchloser kristalliner Feststoff mit saurem Geschmack. Sie ist gut löslich in Wasser und eine starke Säure.

Die L-Form der Weinsäure kommt in vielen Pflanzen und Früchten vor. Sie kann in freier Form sowie als Kalium-, Calcium- oder Magnesium-Salz vorliegen. Im Traubensaft liegt teils freie Weinsäure vor, teils auch Kaliumhydrogentartrat, das sich als Weinstein zusammen mit Calciumtartrat nach der Gärung des Weins abscheidet. D-(–)-Weinsäure ist in der Natur sehr selten. Sie findet sich z. B. in den Blättern des westafrikanischen Baumes *Bauhinia reticulata*. Weinsäure bildet mit Schwermetallionen wie Kupfer, Eisen und Blei Komplexe (z. B. Fehlingsche Lösung zur Bestimmung von Zucker im Urin) (RÖMPP-Redaktion und Hartmann-Schleier 2007).

Weinsäure ist als Lebensmittelzusatzstoff E334 zugelassen und dient als Geschmacksverstärker und Säuerungsmittel z. B. in Speiseeis, Limonaden und Backpulver (Hartwig 2015; RÖMPP-Redaktion und Hartmann-Schleier 2007). Ferner wird sie z. B. in der Galvanotechnik, zur Glasversilberung, zum Metallfärben, zur Herstellung von Brechweinstein und Weichmachern, als Hilfsstoff für Lacke und als Intermediat zur Herstellung anderer Chemikalien eingesetzt. Darüber hinaus findet sie in der Textilindustrie beim Färben und Drucken als Säure und Reduktionsmittel Verwendung (RÖMPP-Redaktion und Hartmann-Schleier 2007).

Der Arbeitsplatzgrenzwert (AGW) für L-(+)-Weinsäure [87-69-4] beträgt 2 mg/m^3^, der Kurzzeitwert ist der Spitzenbegrenzungs-Kategorie I mit dem Überschreitungsfaktor 2 zugeordnet (AGS 2025). In der MAK- und BAT-Werte-Liste hat L-(+)-Weinsäure einen MAK-Wert in gleicher Höhe wie der Arbeitsplatzgrenzwert (DFG 2024). Auch Spitzenbegrenzungs-Kategorie und Überschreitungsfaktor sind gleich. Stoffdaten zur Weinsäure können der Tabelle 1 entnommen werden. Für D-(–)-Weinsäure und *meso*-Weinsäure liegen weder ein AGW noch ein MAK-Wert vor.

**Tab.1:** Stoffdaten zu Weinsäure (Huisman et al. 2013; RÖMPP-Redaktion und Hartmann-Schleier 2007)

Name	L-(+)-Weinsäure	D-(–)-Weinsäure	DL-(±)-Weinsäure
CAS-Nr.	87-69-4	147-71-7	133-37-9
Molmasse [g/mol]	150,09	150,09	150,09
Aggregatzustand bei 20 °C	fest	fest	fest
Dichte bei 20 °C [g/cm^3^]	1,76	1,76	1,76
Dampfdruck bei 25 °C [Pa]^a)^	–	–	1,4 × 10^–9^–3,2 × 10^–1^
Schmelzpunkt [°C]	169–170	169–170	205–206
Siedepunkt bei 1013 hPa [°C]	–	–	–
Wasserlöslichkeit [g/l] bei 20 °C	1390	1390	1390
Beurteilungsmaßstäbe			
Deutschland: AGW, MAK-Wert (AGS 2025; DFG 2024)	2 mg/m^3^	–	–
Spitzenbegrenzungs-Kategorie (Überschreitungsfaktor) (AGS 2025; DFG 2024)	I(2)	–	–

a) Aus Huisman et. al. (2007) wurden zehn Dampfdrücke für Weinsäure entnommen, von denen acht Dampfdrücke kleiner oder gleich 1,8 × 10^–4^ Pa sind. Die Dampfdrücke wurden experimentell aus fester und flüssiger Weinsäure sowie rechnerisch mit geeigneten Modellen ermittelt.

## 3 Grundlage des Verfahrens

Im Rahmen der Methodenentwicklung wurde auf ein Racemat (DL-(±)-Weinsäure) zurückgegriffen, so dass mit der vorliegenden Methode die Konzentrationen von L-(+)- bzw. D-(–)-Weinsäure ermittelt werden können. Im Folgenden wird das racemische Gemisch DL-(±)-Weinsäure der Übersichtlichkeit halber lediglich als Weinsäure bezeichnet. Da *meso*-Weinsäure in der Natur praktisch nicht vorkommt, hat sie als Gefahrstoff an Arbeitsplätzen keine Bedeutung und wurde daher in der Methodenentwicklung nicht berücksichtigt. Aufgrund ihrer physikalischen Eigenschaften eluiert sie auf der verwendeten Säule deutlich früher als DL-(±)-Weinsäure (vgl. Abschnitt 10.8).

Mit diesem Analysenverfahren können L-(+)- und D-(–)-Weinsäure in der Luft am Arbeitsplatz in einem Konzentrationsbereich vom 0,1- bis zum 2-Fachen des MAK-Wertes und des derzeit gültigen AGW für L-(+)-Weinsäure von 2 mg/m^3^ bestimmt werden (AGS 2025; DFG 2024). Auch der Kurzzeitwert mit einem Überschreitungsfaktor von 2 kann gemessen werden (AGS 2025; DFG 2024; DIN 2021).

Zur Probenahme wird mit Hilfe einer Probenahmepumpe ein definiertes Luftvolumen aus dem Atembereich durch einen Glasfaserfilter, der sich in einem Gesamtstaubprobenahmekopf (GSP) befindet, gesaugt. Nach Elution und ionenchromatographischer Trennung erfolgt die Detektion des Säureanions (Tartrat) mittels Leitfähigkeitsdetektor. Die quantitative Auswertung für Weinsäure erfolgt anhand zweier Mehrpunktkalibrierungen mit externer Kalibrierung.

## 4 Geräte, Chemikalien und Lösungen

### 4.1 Geräte

Für die Probenahme:

- Pumpe zur personengetragenen Probenahme, Luftvolumenstrom 3,5 l/min (z. B. GilAirplus, Sensidyne, Saint Petersburg, FL, USA, vertrieben durch z. B. Fa. DEHA Haan & Wittmer GmbH, 71296 Heimsheim)
- Personengetragenes Gefahrstoff-Probenahmesystem (PGP) mit Probenahmekopf GSP für die einatembare Staubfraktion mit Ansaugkegel 3,5 l/min und geeigneter Filterkassette (z. B. Fa. DEHA Haan & Wittmer GmbH, 71296 Heimsheim)
- Massendurchflussmesser 0–20 l/min (z. B. TSI 4146, Fa. TSI GmbH, 52068 Aachen)
- Glasfaserfilter, bindemittelfrei, Durchmesser 37 mm (z. B. MN85/90 BF, Ref: 4060037, Fa. Macherey-Nagel GmbH & Co. KG, 52355 Düren)

Für die Probenvorbereitung und analytische Bestimmung:

- Analysenwaage (z. B. XPE205 Delta Range, Fa. Mettler-Toledo GmbH, 35396 Gießen)
- Edelstahlspatel
- Wägeschiffchen aus Glas
- Messkolben, 10 ml, 25 ml, 100 ml, 250 ml und 2000 ml (z. B. Fa. Brand GmbH + Co. KG, 97877 Wertheim)
- Variable Kolbenhubpipetten mit 10–100 µl und 100–1000 µl Volumen (z. B. Reference 2, Fa. Eppendorf AG, 22366 Hamburg)
- Vollpipette, 10 ml (z. B. Fa. Brand GmbH + Co. KG, 97877 Wertheim) mit Pipettierhilfe
- Edelstahlpinzette
- Braunglasflaschen, 10 ml mit Teflondichtung (z. B. Dichtscheibe G18, CS-Chromatographie Service GmbH, 52379 Langerwehe)
- Dispenser, 10 ml (z. B. Dispensette, Fa. Brand GmbH + Co. KG, 97877 Wertheim)
- Laborschüttler (z. B. Schüttler KS 15 A Control, Fa. Edmund Bühler GmbH, 72379 Hechingen)
- Einmalspritzen aus Polyethylen (PE), 10 ml (z. B. BD Discardit II, Fa. Becton Dickinson and Company, Franklin Lakes; NJ, USA)
- Einmalkanülen, 1,2 × 40 mm (z. B. BD Microlance 3, Fa. Becton Dickinson and Company, Franklin Lakes, NJ, USA)
- PTFE-Einmalfilter mit Luer-Ausgang, Durchmesser 25 mm, Porenweite 0,45 µm (z. B. Chromafil Xtra H-PTFE-45/25, Ref: 729246, Fa. Macherey-Nagel GmbH & Co. KG, 52355 Düren)
- Autosamplergefäße aus PE, 2,5 ml mit perforiertem Stopfen (z. B. Art-Nr. 6.2743.040 und Art-Nr. 6.2743.077, Fa. Metrohm Deutschland GmbH & Co. KG, 70794 Filderstadt)
- Ionenchromatograph mit Entgaser, Säulenofen, Autosampler, chemischer und CO_2_-Suppression, Leitfähigkeits- und UV-Detektor (z. B. 930 Compact IC Flex, Fa. Metrohm Deutschland GmbH & Co. KG, 70794 Filderstadt)
- Trennsäule Metrosep A Supp 16-250/4.0 mit Vorsäule Metrosep A Supp 16 Guard/4.0 (z. B. Art-Nr. 6.1031.430 und Art-Nr. 6.1031.500, Fa. Metrohm Deutschland GmbH & Co. KG, 70794 Filderstadt)

### 4.2 Chemikalien

- Weinsäure, wasserfrei, ≥ 99,5 % (z. B. Carl Roth GmbH + Co. KG, 76185 Karlsruhe)
- Natriumcarbonat, wasserfrei, p. a., ≥ 99,9 % (z. B. Art.-Nr. 1.06392.1000, Fa. Merck KGaA, 64271 Darmstadt)
- Natriumhydrogencarbonat, p. a., ≥ 99,5 % (z. B. Art.-Nr. 1.06329.1000, Fa. Merck KGaA, 64271 Darmstadt)
- Schwefelsäure, 2,5 mol/l (5 N) in wässriger Lösung (z. B. AVS TITRINORM volumetrische Lösung, zur Suppressor-Regeneration, Art.-Nr. 30138293, Fa. VWR International, 94126 Fontenay-sous-Bois, Frankreich)
- Reinstwasser (ρ ≥ 18,2 MΩ × cm bei 25 °C)

### 4.3 Lösungen

Die folgenden Lösungen wurden mit den Chemikalien, welche in Abschnitt 4.2 aufgelistet sind, hergestellt:

**Eluent-Stammlösung:** (1,1 mol Natriumcarbonat/l und 0,5 mol Natriumhydrogencarbonat/l in Reinstwasser)

In einen 100-ml-Messkolben werden 11,6589 g Natriumcarbonat (wasserfrei) und 4,2005 g Natriumhydrogencarbonat gegeben. Danach wird der Messkolben mit Reinstwasser bis zur Marke aufgefüllt und geschüttelt.

**Eluent** (IC-Eluent und zur Elution der Glasfaserfilter): (5,5 mmol Natriumcarbonat/l und 2,5 mmol Natriumhydrogencarbonat/l in Reinstwasser)

In einen 2000-ml-Messkolben, in dem ca. 500 ml Reinstwasser vorgelegt wurden, werden 10 ml der Eluent-Stammlösung mit Hilfe einer Vollpipette zugegeben. Danach wird der Messkolben mit Reinstwasser bis zur Marke aufgefüllt und geschüttelt.

**Weinsäure-Stammlösung 1** (für die Kalibrierung): (40 mmol/l in Reinstwasser)

In einen 100-ml-Messkolben werden 603,38 mg Weinsäure (99,5 % Reinheit) genau eingewogen. Danach wird der Messkolben mit Reinstwasser bis zur Marke aufgefüllt und geschüttelt. Die Konzentrationen der Weinsäure-Stammlösung 1 beträgt 6,0036 g/l bzw. 40 mmol/l.

**Weinsäure-Stammlösung 2** (für die Kontrollstandards): (20 mmol/l in Reinstwasser)

In einen 100-ml-Messkolben werden 301,69 mg Weinsäure (99,5 % Reinheit) genau eingewogen. Danach wird der Messkolben mit Reinstwasser bis zur Marke aufgefüllt und geschüttelt. Die Konzentrationen der Weinsäure-Stammlösung 2 beträgt 3,0018 g/l bzw. 20 mmol/l.

Die Stammlösungen sind lichtgeschützt bei Raumtemperatur mindestens vier Wochen haltbar.

### 4.4 Kalibrier- und Kontrollstandards

**Kalibrierstandards:**

Aus der Weinsäure-Stammlösung 1 werden zwölf Kalibrierlösungen wie folgt hergestellt:

In fünf 10-ml-Messkolben, die als Vorlage je ca. 5 ml Eluent enthalten, werden für den unteren Konzentrationsbereich die in Tabelle 2 aufgeführten Volumina an Weinsäure-Stammlösung 1 zudosiert. Analog dazu werden für den oberen Konzentrationsbereich in sieben 25-ml-Messkolben jeweils die in Tabelle 3 aufgeführten Volumina der Weinsäure-Stammlösung 1 gegeben. Die Messkolben werden mit Eluent bis zur Marke aufgefüllt und geschüttelt. Die Konzentrationen der Kalibrierstandards sind in den Tabellen 2 und 3 aufgeführt.

**Tab.2:** Pipettierschema zur Herstellung der fünf Kalibrierstandards von Weinsäure im unteren Konzentrationsbereich

Kalibrierstandard	Weinsäure-Stammlösung 1 [µl/10 ml]	Konzentration [mg/l]
I	10	6,004
II	30	18,01
III	50	30,02
IV	70	42,03
V	90	54,03

**Tab.3:** Pipettierschema zur Herstellung der sieben Kalibrierstandards von Weinsäure im oberen Konzentrationsbereich

Kalibrierstandard	Weinsäure-Stammlösung 1 [µl/25 ml]	Konzentration [mg/l]
VI	200	48,03
VII	300	72,04
VIII	400	96,06
IX	500	120,1
X	600	144,1
XI	700	168,1
XII	800	192,1

Die Kalibrierstandards überdecken bei 0,42 m^3^ Probeluftvolumen und 10 ml Eluatvolumen etwa 7 bis 230 % des AGWs.

**Kontrollstandards:**

In 10-ml- bzw. 25-ml-Messkolben, die als Vorlage jeweils ca. 5 ml Eluent enthalten, werden 400 µl bzw. 250 µl Weinsäure-Stammlösung 2, wie in Tabelle 4 aufgeführt, zudosiert. Die Messkolben werden mit Eluent bis zur Marke aufgefüllt und geschüttelt. Die Konzentrationen der Kontrollstandards sind in der Tabelle 4 aufgeführt. Der Kontrollstandard I liegt in der Mitte des unteren Kalibrierbereiches und der Kontrollstandard II in der Mitte des oberen Kalibrierbereichs.

**Tab.4:** Pipettierschema zur Herstellung der Kontrollstandards

Kontrollstandard	Weinsäure-Stammlösung 2 [µl]	Endvolumen [ml]	Konzentration [mg/l]
I	250	25	30,02
II	400	10	120,1

## 5 Probenahme und Probenaufbereitung

### 5.1 Vorbereitung der Probenträger

Eine spezielle Vorbereitung der eingesetzten Glasfaserfilter ist nicht notwendig. Zur Vorbereitung der Probenträger wird in die Filterkassette zunächst ein Stützgitter und darauf ein Glasfaserfilter gelegt. Die Filterkassette bleibt bis zur Probenahme mit den dafür vorgesehenen Deckeln verschlossen.

Die eingesetzte Charge an Glasfaserfiltern ist auf einen möglichen Blindwert zu prüfen.

### 5.2 Probenahme

Zur Probenahme wird die mit einem Stützgitter und einem Glasfaserfilter bestückte Filterkassette in den GSP-Probenahmekopf (Riediger 2001) eingesetzt und mit dem Ansaugkegel für 3,5 l/min ausgestattet. Als Probenahmedauer können 15 Minuten zur Messung des Kurzzeitwerts oder 2 Stunden zur Messung des Schichtmittelwertes gewählt werden, woraus ein Probeluftvolumen von 52,5 Litern bzw. 420 Litern resultiert. Die Probenahme kann personengetragen oder ortsfest erfolgen. Nach Beendigung der Probenahme ist der Volumenstrom auf Konstanz zu überprüfen. Ist die Abweichung vom eingestellten Volumenstrom größer als ± 5 %, wird empfohlen, die Probe zu verwerfen (DIN 2023). Die Filterkassette mit dem beaufschlagten Filter wird mit den dafür vorgesehenen Deckeln verschlossen und ins Labor transportiert.

Zu jeder Probenserie ist eine Blindprobe („Field Blank“) mitzuführen. Diese unterscheidet sich von der Analysenprobe lediglich darin, dass keine Probeluft durch den Filter gesaugt wurde. Die Blindprobe wird analog den Proben gelagert und aufbereitet.

### 5.3 Probenaufbereitung

Spätestens 24 Stunden nach der Probenahme wird der Filter vorsichtig mit einer Pinzette aus der Filterkassette entnommen, in eine 10-ml-Braunglasflasche überführt, mittels Dispenser mit 10 ml IC-Eluent überschichtet und kurz manuell geschüttelt. Durch den alkalischen pH-Wert der Elutionslösung liegt Weinsäure als Säureanion (Tartrat) vor. Dieses wird bei der Chromatographie als Analyt bestimmt. Die verschlossene Flasche wird bis zur Analyse bei 2 bis 8 °C im Kühlschrank gelagert.

Zur Probenaufbereitung wird die Braunglasflasche mit dem beaufschlagten Filter aus dem Kühlschrank entnommen und auf Raumtemperatur akklimatisiert. Danach schließt sich eine Behandlung im Laborschüttler für 60 Minuten bei 300 Umdrehungen pro Minute an.

Von der Suspension werden ca. 4 ml Flüssigkeit mit einer Einmalspitze entnommen. Der erste halbe Milliliter, der durch einen Einmalfilter (0,45 µm Porenweite) filtriert wird, wird verworfen. Das nachfolgende Filtrat wird in einem Autosamplergefäß aufgefangen. Die Probeflasche mit der Restflüssigkeit wird verschlossen und als Rückstellprobe im Kühlschrank aufbewahrt.

Die Blindprobe („Field Blank“) wird wie die gesammelten Proben aufbereitet und analysiert.

Es wird empfohlen, zusätzlich einen Reagenzienblindwert („Lab Blank“) zu bestimmen.

## 6 Instrumentelle Arbeitsbedingungen

**Table:** 

**Gerät:**	Ionenchromatograph mit Entgaser, Säulenofen, Autosampler, chemischer und CO_2_-Suppression
**Vorsäule:**	MetroSep A Supp 16 Guard/4.0
**Trennsäule:**	MetroSep A Supp 16-250/4.0
**Säulentemperatur:**	50 °C
**Detektor:**	Leitfähigkeitsdetektor
**Mobile Phase:**	5,5 mmol Natriumcarbonat und 2,5 mmol Natriumhydrogencarbonat, isokratisch
**Flussrate:**	0,75 ml/min
**Injektionsvolumen:**	10 µl
**Laufzeit:**	40 min

Unter den angegebenen Bedingungen hat Tartrat eine Retentionszeit von ca. 24,5 Minuten.

## 7 Analytische Bestimmung

Zur analytischen Bestimmung werden jeweils 10 µl der nach Abschnitt 5.3 aufbereiteten Proben in den Ionenchromatographen injiziert und unter den in Abschnitt 6 angegebenen Bedingungen analysiert. Je nach Höhe der Konzentration an Weinsäure in einer Probe wird zur Auswertung die Kalibrierkurve im niedrigen oder hohen Konzentrationsbereich herangezogen. Liegen die ermittelten Konzentrationen oberhalb des Kalibrierbereiches, so sind geeignete Verdünnungen mit dem Eluent herzustellen und diese nochmals zu analysieren. Des Weiteren werden die aufbereitete Blindprobe („Field Blank“) und der Reagenzienblindwert („Lab Blank“) analog den Analysenproben analysiert.

## 8 Kalibrierung

Externe Kalibrierung:

Zur Erstellung der Kalibrierfunktionen werden die unter Abschnitt 4.4 beschriebenen Kalibrierstandards entsprechend den Abschnitten 6 und 7 analysiert und die ermittelten Peakflächen gegen die jeweiligen Konzentrationen aufgetragen.

Die Kalibrierfunktionen sind im untersuchten Konzentrationsbereich linear und sollten in der Routineanalytik arbeitstäglich überprüft werden. Dazu ist bei jeder Analysenreihe ein Kontrollstandard bekannter Konzentration zu analysieren.

Haben sich die analytischen Bedingungen geändert oder gibt die Qualitätskontrolle Anlass dazu, ist eine neue Kalibrierung zu erstellen.

## 9 Berechnung des Analysenergebnisses

Unter Berücksichtigung des Probeluftvolumens, des Eluatvolumens, der Verdünnung und der Wiederfindung wird die Konzentration von Weinsäure in der Luft am Arbeitsplatz gemäß Gleichung 1 berechnet. Wenn eine Wiederfindung von 100 ± 5 % im Bereich von einem Zehntel bis zum Doppelten des Grenzwertes ermittelt wurde, ist keine Korrektur in Gleichung 1 vorzunehmen.


(1)

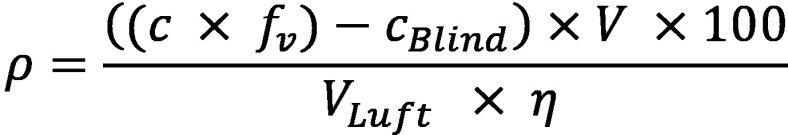



Es bedeuten: 

**Table:** 

*ρ*	Massenkonzentration von Weinsäure in der Luftprobe in mg/m^3^
*c*	Konzentration von Weinsäure in der Messlösung in mg/l
*f*_v_	Verdünnungsfaktor
*c_Blind_*	Konzentration des Field Blanks (Mittelwert) in mg/l
*V*	Volumen des Eluats in Liter (hier 0,01 Liter)
*V_Luft_*	Probeluftvolumen in m^3^ (ermittelt aus Volumenstrom und Probenahmedauer, hier bei 2-stündiger Probenahme 0,42 m^3^)
*ƞ*	Wiederfindung in %

## 10 Beurteilung des Verfahrens

Die Kenndaten der Methode wurden gemäß DIN EN 482 (DIN 2021) sowie DIN 32645 (DIN 2008) ermittelt. Es wurde eine vollständige Validierung der Methode durchgeführt.

### 10.1 Wiederholpräzision

Zur Ermittlung der Wiederholpräzision wurde an sechs Tagen je ein Kalibrierstandard im mittleren Konzentrationsbereich der Kalibriergeraden analysiert. Die ermittelte relative Standardabweichung betrug im mittleren Bereich der unteren Kalibriergerade (30,18 mg/l) 0,94 % und im mittleren Bereich der oberen Kalibriergerade (121,7 mg/l) 0,67 %.

### 10.2 Wiederfindung und Vergleichspräzision

Für die Ermittlung der Wiederfindung wurden je sechs Filter mit drei unterschiedlichen Gehalten an Weinsäure durch Dotierung mit Weinsäurelösung belegt.

Die Dotierlösung 1 wurde durch Einwaage von 4218 mg Weinsäure (99,5 % Reinheit) in einen 250-ml-Messkolben, Auffüllen mit Reinstwasser bis zur Marke und nachfolgendem manuellen Schütteln hergestellt. Dotierlösung 1 hatte eine Konzentration von 16 790 mg/l.

Durch Verdünnen der in Tabelle 5 aufgeführten Volumina der Dotierlösung 1 mit Reinstwasser auf 100 ml wurden die Dotierlösungen 2 und 3 erstellt. Dort sind auch die Konzentrationen der Dotierlösungen aufgeführt. Die Verdünnungsfaktoren beziehen sich auf Dotierlösung 1.

**Tab.5:** Herstellung der Dotierlösungen 2 und 3 für die Filterbelegung

**Dotierlösung**	**Verdünnungsfaktor**	**Volumen der Dotierlösung 1 ** **[ml]**	**Volumen ** **[ml]**	**Konzentration ** **[mg/l]**
2	2	50	100	8394
3	20	5	100	839,4

Die Filter wurden jeweils gemäß Tabelle 6 mit 100 µl der Dotierlösungen 1 bis 3 belegt, was den Konzentrationen bei einem Zehntel des AGWs, beim AGW bzw. dem Doppelten des AGWs bei einem Probeluftvolumen von 0,42 m^3^entspricht.

**Tab.6:** Pipettierschema zur Herstellung der dotierten Filter und Wiederfindungen

**Konzentration entsprechend^a)^**	**Dotierlösung**	**Volumen Dotierlösung** **[µl]**	**Gehalt pro Filter** **[mg]**	**Wiederfindung ** **[%]**	**Standardabweichung (rel.)** **[%]**
0,1 AGW	3	100	0,08395	103,8	0,63
1 AGW	2	100	0,8395	100,5	2,89
2 AGW	1	100	1,679	100,1	0,57

a) bei 10 ml Eluatvolumen und 0,42 m^3^ Probeluftvolumen

Nach dem Trocknen wurden 0,42 m^3^ Laborluft mit einem Volumenstrom von 3,5 l/min bei 20 °C Raumtemperatur und 50 % relativer Feuchte durch die dotierten Filter gesaugt. Anschließend wurden die Filterkassetten mit Deckeln verschlossen und die Filter am darauffolgenden Tag gemäß Abschnitt 5.3 aufbereitet.

Die analytische Bestimmung wurde gemäß den Abschnitten 6 und 7 durchgeführt. Die Berechnung der Konzentrationen entsprechend 1 AGW und 2 AGW erfolgte im Gegensatz zu den Konzentrationen entsprechend 0,1 AGW mit der Kalibriergeraden im oberen Konzentrationsbereich.

Die Wiederfindung für alle drei Konzentrationen ist in Tabelle 6 dargestellt.

Die mittlere Wiederfindung betrug für Weinsäure 101,5 %.

### 10.3 Kapazität des Probenträgers

Versuche mit drei dotierten Filtern entsprechend 1 AGW und 0,84 m^3^ Probeluftvolumen – entsprechend einer vierstündigen Probenahmedauer bei 3,5 l/min Luftvolumenstrom – zeigten mit durchschnittlich 99,9 % eine Wiederfindung in gleicher Höhe.

Die Wiederfindung muss bei der Berechnung der Analysenergebnisse auch bei einer vierstündigen Probenahme und einer Luftkonzentration in der Höhe des AGWs nicht berücksichtigt werden.

### 10.4 Erweiterte Messunsicherheit des Gesamtverfahrens

Zur Ermittlung der erweiterten Messunsicherheit wurden jeweils sechs Glasfaserfilter mit unterschiedlichen Weinsäure-Massen wie in Abschnitt 10.2 beschrieben dotiert, getrocknet und allen Schritten der Probenvorbereitung und Analytik gemäß den Abschnitten 5.3, 6 und 7 unterworfen. Die Filterbelegung entsprach damit den Konzentrationen von einem Zehntel des AGWs, des AGWs bzw. dem Doppelten des AGWs bei 0,42 m^3^ Probeluftvolumen.

Die erweiterte Messunsicherheit wurde unter Berücksichtigung aller relevanten Einflussgrößen nach DIN EN 482 (DIN 2021) und DIN EN ISO 21832 (DIN 2020) abgeschätzt und mit Hilfe des IFA-Excel-Sheets (IFA o.J.) zur Berechnung der erweiterten Messunsicherheit berechnet.

Die Kombination aller Unsicherheitsbeiträge führt zu den konzentrationsabhängigen kombinierten Messunsicherheiten. Durch Multiplikation mit dem Wahrscheinlichkeitsfaktor k = 2 (für 95 % Sicherheit) werden die entsprechenden konzentrationsabhängigen erweiterten Messunsicherheiten des Gesamtverfahrens erhalten.

Tabelle 7 fasst alle ermittelten Unsicherheitsbeiträge zusammen, wobei zwischen geringer, mittlerer und hoher Filterbelegung unterschieden wird. Geringe, mittlere und hohe Konzentration entsprechen einem Zehntel des AGWs, dem AGW bzw. dem Doppelten des AGWs. Die Messunsicherheiten wurden für die Probenahmedauern von 15 Minuten und 120 Minuten berechnet.

**Tab.7:** Bestimmung der Messunsicherheit; Angaben in %

**Unsicherheit**	**15 Minuten Probenahmedauer**	**120 Minuten Probenahmedauer**
*u* Probenahme, Transport, Lagerung	9,3	8,9
*u* Wiederfindung geringe Konzentration	4,0	4,0
*u* Wiederfindung mittlere Konzentration	1,6	1,6
*u* Wiederfindung hohe Konzentration	1,5	1,5
*u* analytische Variabilität	3,4	3,5
*U* erweitert geringe Konzentration	21,4	20,8
*U* erweitert mittlere Konzentration	20,1	19,4
*U* erweitert hohe Konzentration	20,1	19,4

Für das beschriebene Messverfahren ergeben sich erweiterte Messunsicherheiten von 19 bis 21 % für den Messbereich von einem Zehntel bis zum Doppelten des Grenzwertes bei einer 2 Stunden dauernden Probenahme. Die erweiterten Messunsicherheiten für den Messbereich der Hälfte bis zum Doppelten des Kurzzeitwertes bei einer 15 Minuten dauernden Probenahme liegen zwischen 20 und 21 %.

### 10.5 Einfluss der Luftfeuchte

Der Einfluss der Luftfeuchte wurde zusätzlich bei 20 % und 80 % relativer Feuchte und 21 °C Lufttemperatur geprüft. Die Wiederfindungen der Weinsäure weisen in diesem Bereich keine Abhängigkeit von der relativen Luftfeuchte auf.

Die relative Feuchte muss zwischen 20 und 80 % und bei einer Probenahmetemperatur von ca. 21 °C bei der Berechnung der Analysenergebnisse nicht berücksichtigt werden. Sollten die Umgebungsbedingungen davon abweichen, ist das Messverfahren dahingehend zu überprüfen.

### 10.6 Bestimmungsgrenze

Die Ermittlung der Bestimmungsgrenze erfolgte aus einer äquidistanten 10-Punkt-Kalibrierung gemäß DIN 32645 (DIN 2008) im unteren Konzentrationsbereich. Die ermittelte absolute Bestimmungsgrenze beträgt 0,018 mg/l.

Die 10-Punkt-Kalibrierung wurde durch Einwaage von Weinsäure und mehrfache Verdünnung wie folgt hergestellt:

In einen 100-ml-Messkolben wurden 600,98 mg Weinsäure eingewogen, der Kolben mit Reinstwasser wurde bis zur Marke aufgefüllt und geschüttelt. Unter Berücksichtigung der Reinheit betrug die Weinsäurekonzentration 5980 mg/l (Lösung 1).

In einen 250-ml-Messkolben, der ca. 50 ml Eluent enthielt, wurden 750 µl der Lösung 1 gegeben und anschließend bis zur Marke mit Eluent aufgefüllt und geschüttelt. Die Konzentration dieser Weinsäurelösung betrug 17,94 mg/l (Lösung 2).

Es wurden zehn 25-ml-Messkolben vorgelegt, die jeweils 5 ml Eluent enthielten. Hierzu wurden Volumina der Lösung 2 gemäß Tabelle 8 gegeben. Anschließend wurden die Kolben mit Eluent bis zur Marke aufgefüllt und geschüttelt. Tabelle 8 enthält die Konzentrationen der zehn Kalibrierstandards.

**Tab.8:** Pipettierschema zur Herstellung der zehn Kalibrier-Standardlösungen für die Ermittlung der Bestimmungsgrenze

Kalibrierstandard	Volumen Lösung 2 [µl]	Endvolumen [ml]	Konzentration Weinsäure [mg/l]
I	100	25	0,0718
II	140	25	0,1005
III	180	25	0,1292
IV	220	25	0,1579
V	260	25	0,1866
VI	300	25	0,2153
VII	340	25	0,2440
VIII	380	25	0,2727
IX	420	25	0,3014
X	460	25	0,3301

Für Weinsäure ergab sich bei einem Vertrauensbereich von 95 % eine Bestimmungsgrenze von 0,018 mg/l. Unter Berücksichtigung eines Eluatvolumens von 10 ml und eines Probeluftvolumens von 0,42 m^3^ entspricht dies einer relativen Bestimmungsgrenze von 0,00043 mg/m^3^ (Schichtmittelwert). Bezogen auf ein Probeluftvolumen von 0,0525 m^3^ liegt sie bei 0,0034 mg/m^3^ (Kurzzeitwert).

### 10.7 Lagerfähigkeit

Analog der Wiederfindung gemäß Abschnitt 10.2 wurden je 15 Filter entsprechend 0,1 AGW, 1 AGW und 2 AGW, wie in Tabelle 6 aufgeführt, dotiert.

Nach Trocknung an der Luft wurden 0,42 m^3^ saubere Luft mit einem Volumenstrom von 3,5 l/min durch die Filter gesaugt und anschließend die Filterkassetten mit Deckeln verschlossen. Am folgenden Tag wurden die Filter mit der Pinzette entnommen, in 10-ml-Braunglasfaschen überführt und mittels Dispenser mit 10 ml IC-Eluent überschichtet und verschlossen. Nach kurzem manuellem Schütteln wurden die Braunglasflaschen im Kühlschrank bei ca. + 5 °C aufbewahrt. Am Analysetag wurden die Flaschen aus dem Kühlschrank entnommen, auf Raumtemperatur gebracht und gemäß den Abschnitten 5.3, 6 und 7 aufbereitet und analysiert.

Je drei Filter pro Konzentration wurden am Tag der Dotierung (ohne Aufbewahrung im Kühlschrank), nach einer Woche, nach zwei Wochen, nach drei Wochen und nach vier Wochen gemäß Abschnitt 5.3 aufbereitet und am selben Tag gemessen.

Die Lagerdauern von einer Woche, zwei und drei Wochen hatten keinen nennenswerten Einfluss auf die Wiederfindung Die Wiederfindungen nach vier Wochen Lagerung im Kühlschrank sind in Tabelle 9 aufgeführt.

**Tab.9:** Ermittelte Wiederfindungen nach vier Wochen Lagerung im Kühlschrank bei ca. 5 °C

Weinsäuregehalt pro Filter [mg]	Entspricht AGW^a)^	Wiederfindung [%]
0,08395	0,1	94,3
0,8395	1	97,6
1,679	2	99,3

a) bei 10 ml Eluatvolumen und 0,42 m^3^ Probeluftvolumen

Die mittlere Wiederfindung betrug nach vier Wochen Lagerung im Kühlschrank 97,1 %. Die Lagerfähigkeit der beaufschlagten und am Folgetag der Beaufschlagung eluierten Glasfaserfilter ist bei Lagerung im Kühlschrank bei ca. 5 °C 4 Wochen lang gewährleistet.

### 10.8 Selektivität

Es existieren drei Stereoisomere der Weinsäure. Mit der vorliegenden Methode können die Konzentrationen von L-(+)- und D-(–)-Weinsäure bestimmt werden, deren Retentionszeiten identisch sind. Bei gleicher Konzentration sind auch die Messsignale identisch. Im Rahmen der Methodenentwicklung wurde auf ein Racemat (DL-(±)-Weinsäure) zurückgegriffen.

Das Messverfahren wurde für *meso*-Weinsäure [147-73-9] aufgrund deutlich geringerer Retentionszeit bei denselben Messbedingungen und der nicht vorhandenen Relevanz der Verbindung als Gefahrstoff an Arbeitsplätzen nicht validiert.

Zwischen Weinsäure und Tartraten (z. B. Alkalisalzen) kann nicht unterschieden werden. Der im Eluenten lösliche Tartratanteil wird ebenfalls erfasst und als Weinsäure ausgegeben. In der Tabelle 10 werden gängige Tartrate mit ihren Wasserlöslichkeiten aufgeführt. Die Wasserlöslichkeit ist bei diesen Tartraten ausreichend groß, so dass Luftkonzentrationen im Bereich des AGWs für Weinsäure überprüft werden können.

**Tab.10:** Wasserlöslichkeit ausgewählter Tartrate (IFA 2024 a, b, c, d, e)

Substanz	CAS-Nr.	Wasserlöslichkeit bei 20 °C [g/l]	Entspricht bei Erreichen der Sättigungskonzentration einer Luftkonzentration von^a)^ [mg/m^3^]
Ammoniumtartrat	3164-29-2	428	8300
Dikaliumtartrat	921-53-9	235,3	3700
Kaliumantimonyltartrat	6535-15-5	55	290
Kaliumhydrogentartrat	868-14-4	6,2	120
Kaliumnatriumtartrat	304-59-6	630	11 000

a) bei 10 ml Eluatvolumen und 0,42 m^3^ Probeluftvolumen, berechnet als Weinsäure

Die Anionen Fluorid, Bromid, Chlorid, Nitrat, Nitrit und Sulfat sowie die Anionen der Dicarbonsäuren Oxalsäure und Adipinsäure stören die Analyse nicht.

Die Anwesenheit von Bernsteinsäure liefert ein falsch positives Signal, weil Tartrat und Succinat coeluieren. Eine Trennung ist bei den hier gewählten chromatographischen Bedingungen nicht möglich.

Für Phosphat und das Anion der Glutarsäure (Glutarat), die beide coeluieren, liegt mit dem Tartrat-Signal keine vollständige Basislinientrennung vor. Dies beeinträchtigt die Analyse von Weinsäure jedoch kaum.

Das Anion der Äpfelsäure (Malat) eluiert zeitlich kurz vor und Malonat (Anion der Malonsäure) zeitlich kurz nach Tartrat, so dass auch hier keine Basislinientrennung vorliegt. Bei gleich hoher Konzentration im Vergleich zur Weinsäure, geht das Signal in beiden Fällen um 70 % zur Basislinie zurück. Für eine gleichkonzentrierte Lösung mit Äpfelsäure und Weinsäure wurde für Weinsäure ein etwa 10%iger Mehrbefund und für eine gleichkonzentrierte Lösung mit Weinsäure und Malonsäure ein etwa 10%iger Minderbefund festgestellt.

**Abb.2:**
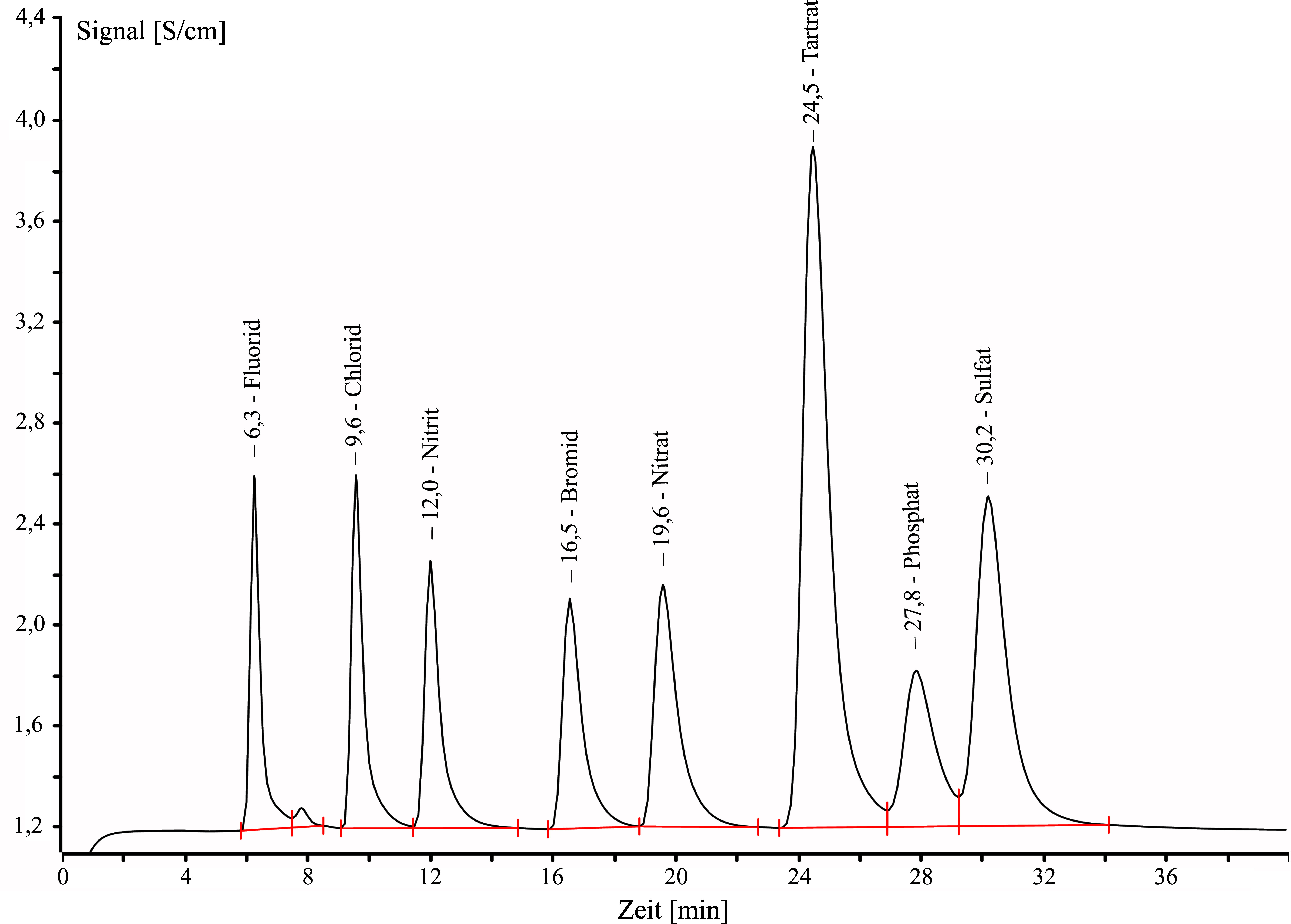
Ionenchromatogramm von Weinsäure (Tartrat, 24,5 min) sowie weiteren Anionen: Fluorid (6,3 min), Chlorid (9,6 min), Nitrit (12,0 min), Bromid (16,5 min), Nitrat (19,6 min), Phosphat (27,8 min), Sulfat (30,2 min). Konzentration der Weinsäure ca. 6 mg/l

Weinsäure ist – wie die zuvor genannten Dicarbonsäuren auch – im UV-Detektor bei einer Wellenlänge von 210 nm optisch aktiv und kann im Bereich der Kalibrierung gemessen werden. Die Bestimmungsgrenze liegt für Weinsäure bei einer Messwellenlänge von 210 nm und einem Vertrauensbereich von 95 % bei ca. 2,6 mg/l. Unter Berücksichtigung eines Eluatvolumens von 10 ml und eines Probeluftvolumens von 0,42 m^3^ entspricht dies einer relativen Bestimmungsgrenze von 0,062 mg/m^3^ (Schichtmittelwert). Die Absorption im UV-Detektor kann als zusätzliches qualitatives Identifizierungsmerkmal für Weinsäure, z. B. als Abgrenzung zum nicht UV-aktiven Phosphat, genutzt werden.

Blindwerte werden durch die parallel zur Probenaufbereitung hergestellten „Field Blanks“ berücksichtigt, konnten allerdings nicht nachgewiesen werden. Die Glasfaserfilter und der Eluent wiesen keine Blindwerte auf.

## 11 Diskussion

Das Messverfahren ermöglicht die Bestimmung von L-(+)- bzw. D-(–)-Weinsäure in der Luft am Arbeitsplatz in einem Konzentrationsbereich von einem Zehntel bis zum Doppelten des derzeit gültigen AGW von 2 mg/m^3^. Das Messverfahren ist auch geeignet, um die Einhaltung des Kurzzeitwertes zu überprüfen. Aufgrund des geringen Dampfdruckes von Weinsäure (vgl. Tabelle 1) ist ein Einfluss der Temperatur bis 40 °C nicht zu erwarten. 

Ferner können auch in der Luft am Arbeitsplatz partikelförmig vorliegende lösliche Tartrate, angegeben als Weinsäure, bestimmt werden.
